# Unravelling the activity rhythms of urban vector mosquitoes with smart-trap technology

**DOI:** 10.1038/s41598-026-38795-y

**Published:** 2026-02-14

**Authors:** María I. González-Pérez, Catuxa Cerecedo-Iglesias, Alex Richter-Boix, Laura Barahona, Tomás Montalvo, John R. B. Palmer, Frederic Bartumeus

**Affiliations:** 1 Department of Ecology and Complexity, Centre d’Estudis Avançats de Blanes (CEAB-CSIC), 17300 Girona, Spain; 2 Agència de Salut Pública de Barcelona (ASPB), 08023 Barcelona, Spain; 3 Universitat Pompeu Fabra, 08002 Barcelona, Spain; 4 ICREA, Institució Catalana de Recerca i Estudis Avançats, 08010 Barcelona, Spain; 5 CREAF, Ecological and Forestry Applications Research Centre, 08193 Barcelona, Spain

**Keywords:** Ecology, Ecology, Environmental sciences, Zoology

## Abstract

Understanding mosquito activity in dense urban areas is essential to assess human exposure to nuisance and health risks. We analyzed real-time mosquito data from four smart traps operating between 2021 and 2024 in Barcelona (NE Spain), focusing on the fine-scale temporal dynamics of two major urban vector species, *Aedes albopictus* and *Culex pipiens*. Both species exhibited consistent bimodal diel activity patterns aligned with sunrise and sunset, with species-specific differences in peak intensity and timing as well as seasonal fluctuations. Using a random forest framework, we identified light-related cues as primary activators of mosquito host-seeking activity; and light cues, temperature and rainfall also acting as modulators of activity, roles varying by species and temporal scale. This activator–modulator perspective illustrates how intrinsic circadian rhythms interact with extrinsic environmental drivers to determine mosquito activity across temporal scales. Our findings highlight the ecological value of high-resolution monitoring and the potential of next-generation surveillance tools to support early warning systems and evidence-based vector control in the context of smart cities.

## Introduction

Mosquitoes (Diptera: Culicidae) represent one of the major threats to global health since they act as vectors of several pathogens that cause severe diseases (e.g. malaria, dengue, yellow fever, Zika, West Nile, chikungunya, and others), resulting in hundreds of thousands of human deaths every year^1^. For a long time, these diseases were confined to low-income countries in the Global South and remained largely neglected. However, in the last decades, mosquito-borne diseases (MBDs) have also become a growing concern in northern latitudes, due to the establishment and spread of both local and imported pathogens and vectors^2,3^. Particularly in cities, where most of the world’s human population live, urbanization processes have increased the risk of MBDs outbreaks^4,5^.

In Europe, the two most concerning mosquito vectors are the autochthonous Northern house mosquito (*Culex* (*Culex*) *pipiens* Linnaeus, 1758) and the invasive Asian tiger mosquito (*Aedes*(*Stegomya*) *albopictus* Skuse, 1894). Both are cosmopolitan species with opportunistic feeding behaviour^6–8^, and are responsible for the local transmission of both endemic and imported arboviruses such as West Nile virus and dengue, respectively^9,10^. The recent increase in MBD cases throughout Europe has raised concerns among public health authorities about the need to strengthen MBD surveillance systems^11–13^.

As other living organisms, mosquitoes are subjected to daily and seasonal environmental fluctuations caused by the rotational and translational movements of the Earth around its own axis and around the Sun^14^. As a product of evolution, these organisms have developed an endogenous circadian clock that regulates many physiological and behavioural processes with a 24-hour periodicity. Some of these processes include locomotor/flight activity, mating, host-seeking, and biting behaviour. All these behaviours are strongly related to their capacity to disseminate diseases, the so-called vectorial capacity^15^. The study of the activity patterns of vector mosquitoes is therefore of key importance for the epidemiology of MBDs^16^.

A substantial part of our understanding of the circadian clock of mosquitoes comes from studies on model organisms, particularly *Drosophila melanogaster*^17,18^. The molecular mechanisms underlying this biological clock rely on a series of negative transcription–translation feedback loops, in which clock genes are regulated by the protein products they encode. Although the endogenous circadian clock is driven by internal oscillators that persist (or “free-run”) in the absence of environmental cues, these oscillations are entrained–or synchronised–by specific environmental signals. This synchronisation, mediated by entrainment agents known as *Zeitgebers*, allows the free-running internal rhythm to align with the periodicity of solar days and seasons^15^.

Two of the most important and well-studied *Zeitgebers* for the mosquito circadian clock are light and temperature^14^. Daily activity peaks in mosquitoes are generally timed around sunrise and sunset, which vary seasonally with geographic latitude. Furthermore, as poikilothermic organisms, mosquitoes do not regulate their body temperature internally; instead, it is determined by the surrounding environment. To avoid thermal stress, mosquito activity is restricted to a specific temperature range, becoming impossible beyond critical thermal minima and maxima^19,20^. In some mosquito species, when winter temperatures drop below tolerable thresholds, individuals enter a phase of arrested development known as diapause^21,22^. While diapause is primarily activated by photoperiod, it is also modulated by temperature, diet, and other environmental factors^23^.

The effects of the photoperiod and the temperature on mosquito daily activity patterns have been extensively studied under laboratory conditions for several disease-vector species^24–26^. However, studying mosquito circadian rhythms in natural settings remains challenging due to the superimposition of daily and seasonal environmental fluctuations and the limited availability of efficient monitoring tools. Traditional methods for monitoring adult mosquitoes, such as baited traps or human landing collections, have long been used to monitor the presence and abundance of vectors^27,28^. Nevertheless, their application to studying mosquito activity and behavioural rhythms in the wild remains constrained to limited spatial and temporal scales^29–31^. Conventional methods demand substantial time and human resources, making them impractical for studies requiring coverage across a wide range of temporal scales. A promising and scalable alternative lies in the deployment of next-generation surveillance tools, such as smart-traps, which enable remote and automated mosquito detection and classification^32^. These technologies offer real-time, high-resolution data collection with minimal manual effort, representing a significant advancement for mosquito behaviour research and vector ecology.

In the vanguard of vector surveillance, the Public Health Agency of Barcelona has incorporated, since 2021, 4 smart-traps in outdoor public spaces for the automated classification of mosquitoes, as a complement to the traditional monitoring methods deployed in their routine surveillance plan, aligning with Barcelona smart-city vision^33^. These smart-traps, which are composed by an intelligent sensor (Irideon S.L.) coupled to the entrance of a conventional mosquito suction trap (Biogents AG), automatically classify those mosquitoes that enter the trap by genus and sex with high accuracy^34–37^. Each classification event is also tagged with a time-stamp and information about the trap location, temperature, and humidity values with a 1 s time-resolution.

Leveraging data from novel automated mosquito monitoring stations, this study aims to comprehensively assess the activity rhythms of two of the most widespread vector species in temperate urban regions: *Aedes albopictus* and *Culex pipiens*. Smart-traps, like traditional mosquito traps, are baited with specific chemical lures designed to attract host-seeking females; however, males are often captured as well. Here, we present standardized daily and seasonal activity patterns for both male and female mosquitoes. As our primary focus is on female mosquitoes (responsible for disease transmission), we developed a Random Forest (RF) model to quantify the relative influence and different activation-modulation roles of key environmental drivers (i.e., *Zeitgebers*) on hourly female host-seeking activity. Finally, we used backward predictions from the RF model to estimate how female mosquito activity patterns may have changed over the past two decades.

This study strengthens our understanding of vector ecology in urban settings and illustrates how the deployment of smart-trap networks represents a paradigm shift towards precision public health interventions to mitigate MBDs. This approach modernizes vector surveillance infrastructure to meet the increasing challenges posed by global change. The high temporal resolution provided by next-generation surveillance tools allows for the detection of daily and seasonal peaks in mosquito activity, which is essential for the timely implementation of preventive and control measures. The network of automated mosquito monitoring stations established by the Public Health Agency of Barcelona serves as a pioneering example of a smart-city initiative in the context of vector surveillance. By partially automating its vector surveillance system, the city enhances its capacity to deliver early warning responses in the face of epidemiological threats.

## Results

### Daily and seasonal activity patterns of *Ae.albopictus* and *Cx.pipiens*

**Fig. 1:**
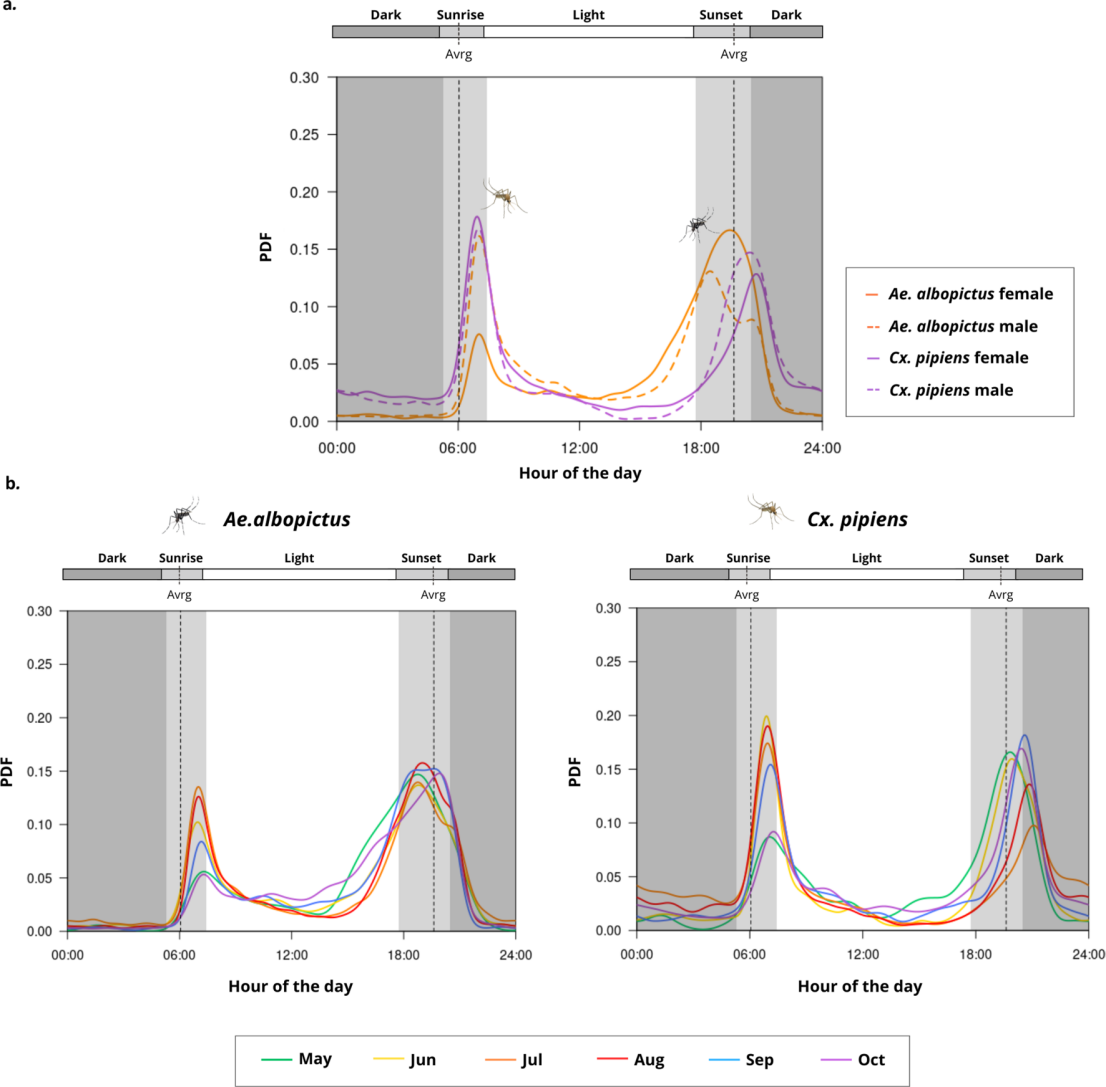
Daily activity peaks of *Ae.albopictus* and *Cx.pipiens* obtained from smart-trap data. Representation of daily mosquito averages: yearly averages by sex and species (**a**), and monthly averages by species (**b**).

Average mosquito daily host-seeking activity deduced from smart-trap data revealed a bimodal activity pattern during the day for the studied species, with two activity peaks concentrated around crepuscular periods (dawn and dusk), as depicted in Fig. 1a. The morning peak occurred shortly after the average sunrise time (indicated by the dashed line), coinciding with the transition from darkness to light, and it was totally time-overlapped for both species and sexes. The activity then decreased during central daylight hours, maintaining low levels until middle afternoon in case of *Ae. albopictus*; and until the evening in case of *Cx.pipiens*. The second activity peak occurred around sunset. However, there was a temporal segregation between both species, with *Ae. albopictus* being more diurnal (with a broader activity peak during daylight hours until dusk) and *Cx.pipiens* being more crepuscular (with a narrower peak concentrated at dusk). During the afternoon peak, males reached their peak of activity slightly earlier than females. In case of *Ae.albopictus*, the activity was mostly concentrated during the afternoon, in contrast to *Cx.pipiens* whose activity was more evenly distributed during the morning and evening peaks, with more emphasis at dawn.

The variation in daily activity patterns across different months (from May to October) for both mosquito species is illustrated in Fig. 1b. In both cases, the bimodal activity pattern remained evident and the sunrise and sunset peaks were consistently present throughout the months, although the intensity and location of the activity peaks varied. In *Ae. albopictus*, the morning peak occurred at the same time for all months during the study period; however, it was higher during summer months (specially in July and August) and was lower during May and October, probably coinciding with the start and the end of mosquito season for this species. The afternoon activity of *Ae. albopictus* was broader and started earlier from cooler to warmer months. The peak of maximum activity during the afternoon was however quite aligned across months and ended up always at the same time. The intensity of the afternoon peak was higher than the morning peak for all months, becoming more evident during May and October. Seasonality similarly affected the daily activity patterns of *Cx. pipiens*. Morning activity peaks occurred consistently at the same time in all months, but their intensity was lower at the beginning (May) and end (October) of the season, probably due to cooler temperatures during those periods. In contrast, the evening peak exhibited a progressive shift to later hours from May to October, although this temporal displacement was clear along the season, it was not consistent month by month. Notably, from June to August, *Cx. pipiens* exhibited higher activity in the morning than in the evening, with a distinctly more pronounced morning peak. This pattern clearly reversed in May and October when the evening peak became more prominent than the morning one.

### Environmental drivers of female mosquito host-seeking activity

We modeled hourly female mosquito activity using a selected subset of environmental variables listed here: hourly temperature, average relative humidity during the last 12 hours, cumulated precipitation during the last 12 hours, distance to sunrise, distance to sunset and daylight hours during a day. The final processed dataset included 17,267 female observations, comprised between the 1st of May and the 31st of October during four consecutive years (2021–2024). Female mosquitoes represented 65.2% of the total samples. The dataset was split 70/30 to train and test the RF model, respectively. A 4-fold cross-validation was applied to the training set, and the best-tuned model was used on the test set for prediction. The final RF model reported a RMSE = 0.26 and a R$^{2}$ = 0.53 for *Ae. albopictus*; and a RMSE = 0.19 and a R$^{2}$= 0.33 for *Cx. pipiens*.

**Fig. 2:**
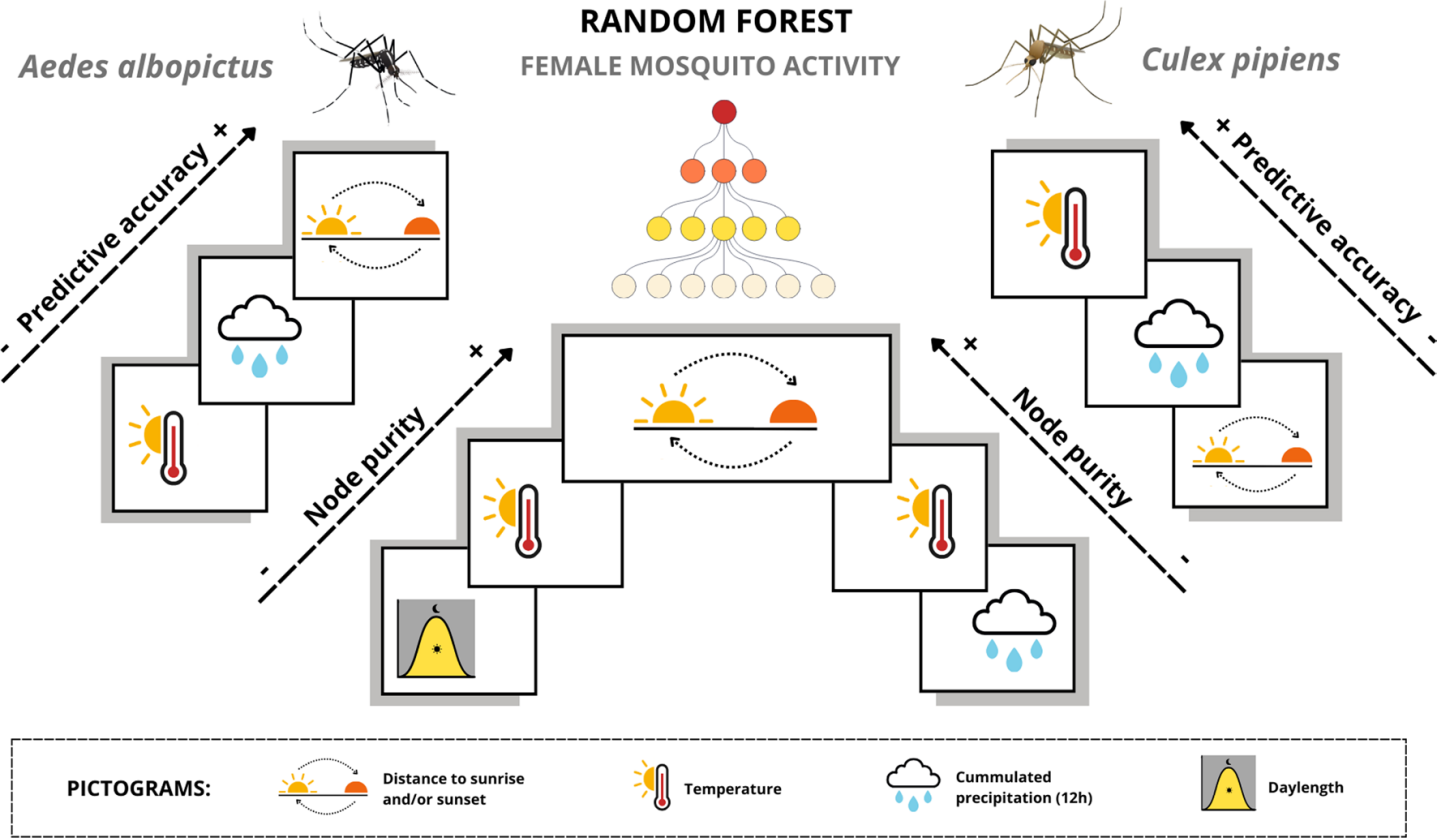
Predictive and structural contributions of the environmental variables to the RF model. From bottom to top, arrows indicate increasing importance of key variables in terms of both predictive accuracy and node purity within the model.

The RF model highlighted some differences between predictive and structural contributions of environmental factors to the model’s performance and its inner structure , respectively (Fig. 2). In *Ae.albopictus*, distance to sunrise and sunset events emerged as the most critical variables for the model’s predictive accuracy, as permuting these variables led to the largest increase in prediction error, indicating that information related to daily photoperiod is highly informative for predicting hourly female activity. Secondarily, model’s predictive accuracy relied on the accumulated precipitation in the last 12 hours and the temperature. The importance of recent precipitation likely reflects a short-term inhibitory effect on host-seeking activity, consistent with the hourly temporal resolution of the response variable. In terms of node purity, the distance to sunrise and sunset, and also the temperature, were the most influential variables, suggesting that they have a main role in partitioning the data and in grouping hours with similar levels of activity female activity within the model structure. For *Cx.pipiens*, the temperature was clearly the most important predictor in terms of predictive accuracy, indicating that thermal conditions provide substantial information for predicting hour-to-hour variation in activity. The accumulated precipitation of the last 12 hours and the distance to sunrise also contributed, to a lesser extent, to explain theobserved activity patterns of this species. As for *Ae. albopictus*, distance to sunrise and sunset were the most influential variables in terms of node purity (followed by temperature), highlighting the importance of daily photoperiod and temperature in structuring the diel distribution of *Cx. pipiens* activity.

See Supplementary Information Fig. S1 to visualize the complete variable importance plots derived from the RF model.

### Long-term trends of female host-seeking activity: backward forecasting

**Fig. 3:**
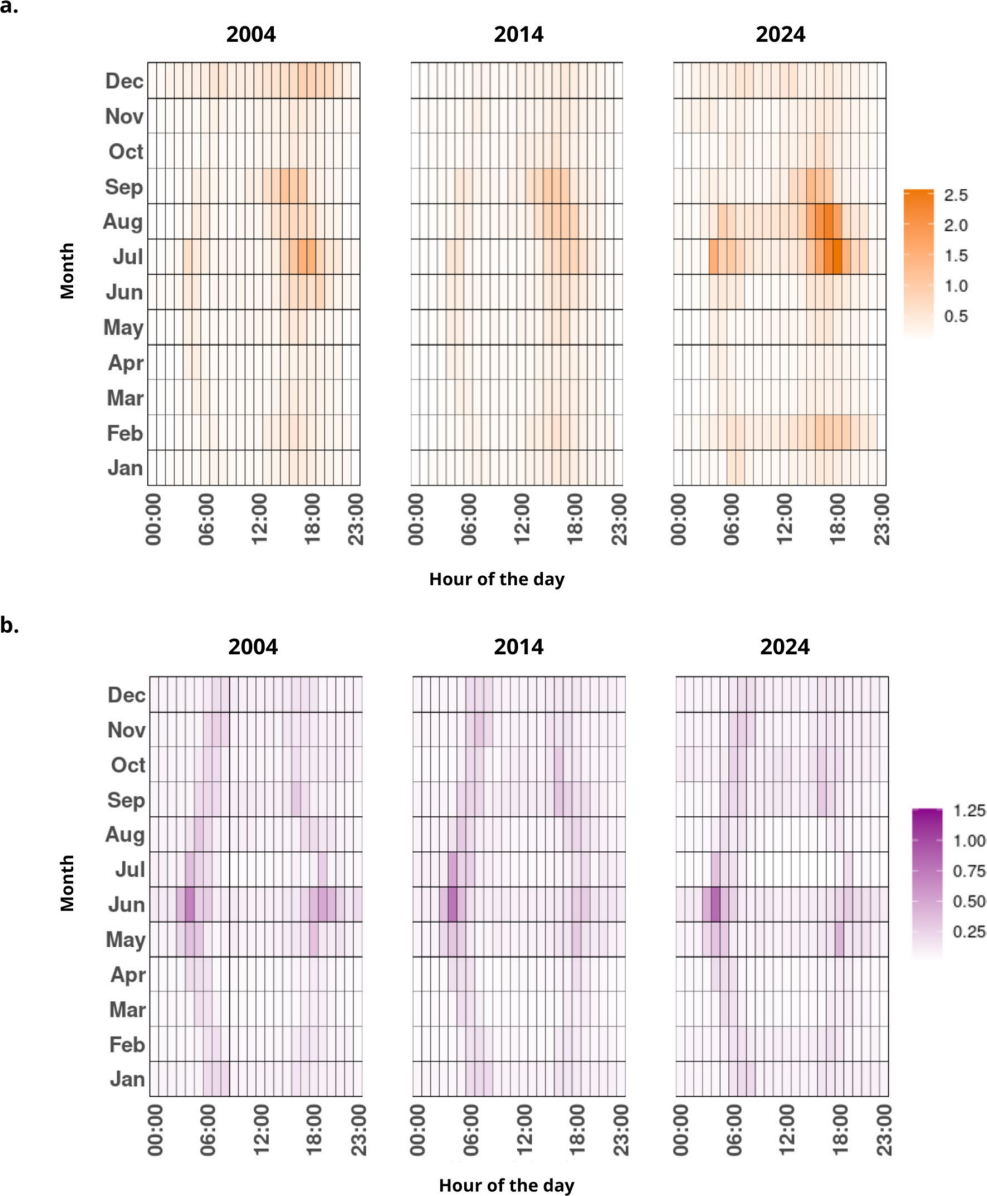
Calendar heatmap showing the predicted historical evolution of female mosquito activity for the species *Ae. albopictus* (**a**) and *Cx.pipiens* (**b**) for the years 2004, 2014 and 2024. The color bar represents the predicted hourly female abundance corrected by sampling effort.

Based on the weather-driven RF model, we predicted the temporal evolution of female mosquito host-seeking activity from 2004 to 2024, at hourly and seasonal scales (Fig. 3).

For *Ae. albopictus* (Fig. 3a), the predicted activity exhibited a pronounced seasonal pattern, with the bulk of activity concentrated between July and September, coinciding with warmer months (see Supplementary Information Fig. S2). A specific diurnal pattern was also evident in the predictions, suggesting a strong preference for daytime activity, which was mainly concentrated in the early afternoon (between 14 and 20 h approximately). Notably, there is an observable increase in the afternoon predicted activity of *Ae. albopictus* over time, being 2024 its maximum exponent (see Supplementary Information Fig. S3 for a complete representation of the evolution of *Ae. albopictus* activity over the past 20 years).

For *Cx. pipiens* (Fig. 3b), the climate-driven RF model predicted a relatively consistent pattern of hourly and seasonal activity across years (see Supplementary Information Fig. S4 for a complete representation of the evolution of *Cx. pipiens* activity over the past 20 years). The highest predicted activity was concentrated during the summer months, particularly in June and July. However, according to model predictions, *Cx. pipiens* remains active all the year, concentrating its activity at specific time-slots (around sunrise and sunset) that vary in time with the advance of the seasons.

To evaluate the coherence of model predictions, we compared the hourly smart-trap observations recorded between May and October 2024 with the corresponding RF-based predictions for the same time period (Supplementary Fig. S5). For both species, the RF model seems to successfully retrieve the dominant seasonal and diel activity signatures although the predicted values appear smoother and somewhat attenuated relative to the observed smart-trap counts. In case of *Ae. albopictus*, the model effectively captures the clear activity increase during summer months (July and August) and the marked afternoon activity peak (between 15 and 19 h). In case of *Cx. pipiens*, the model reproduces its consistent presence throughout all mosquito season and its strong alignment with sunrise and sunset time-slots. Also, the predictions reflect a seasonal shift in diel activity timing for *Cx. pipiens* according to changing photoperiod: during warmer months, activity tends to occur slightly earlier in the morning and later in the evening compared to colder months.

**Fig. 4:**
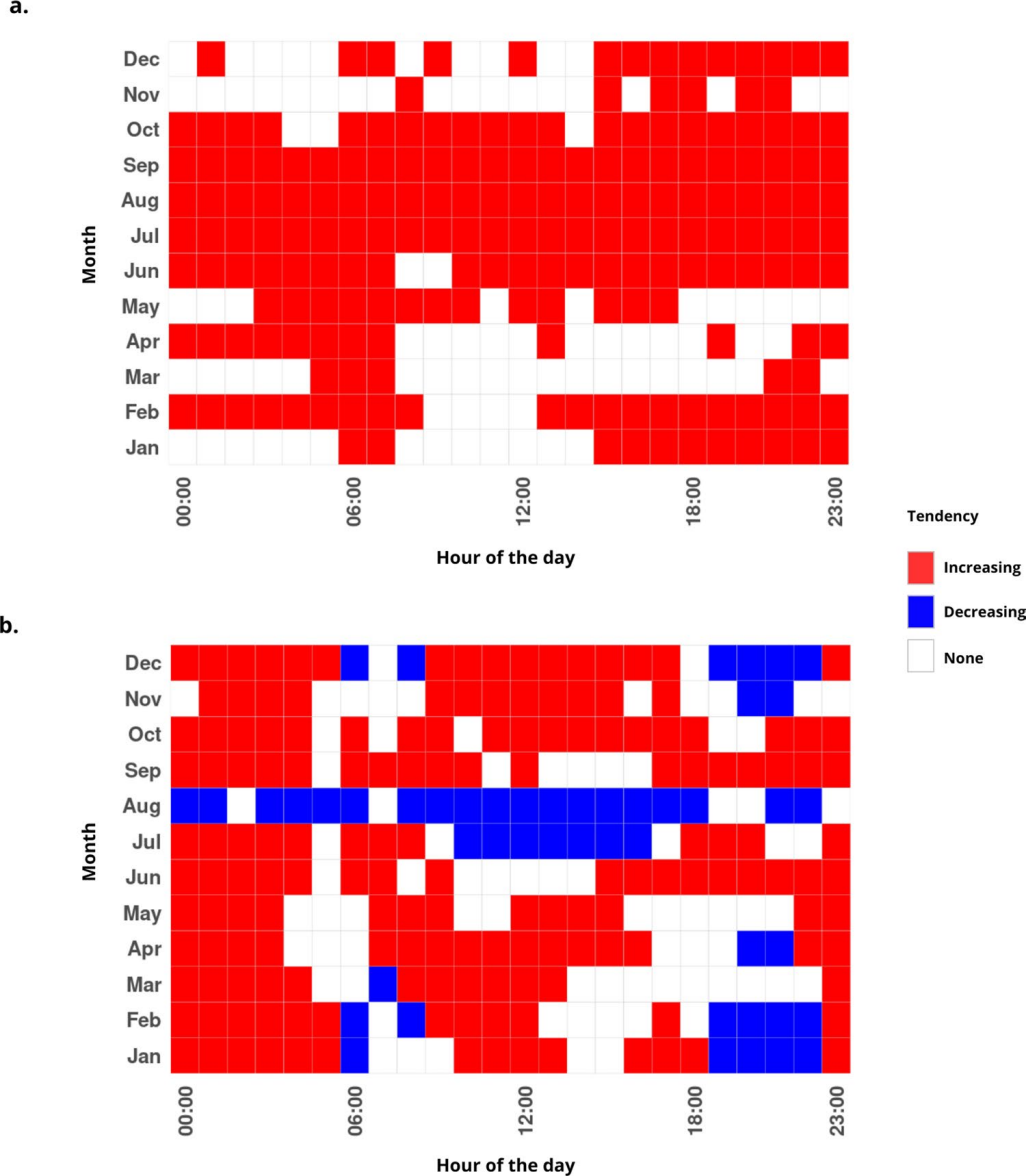
Calendar showing the results from Mann-Kendall test for the temporal trend analysis of activity of female *Ae. albopictus* (**a**) and *Cx. pipiens* (**b**) from 2004 to 2024. P-values < 0.05 indicate that differences found in the hourly female mosquito activity across years were significant, and the corresponding cells were colored in blue (if there was a decreasing trend) and in red (if there was an increasing trend). When there were no significant differences across years (p-value> 0.05), the cells were white-colored.

To quantify whether the predicted hourly activity by RF model exhibited systematic temporal changes over the 2004–2024 period, we applied a Mann–Kendall trend test to each hour–month combination (Fig. 4). This non-parametric test, allows us to detect monotonic increases or decreases in activity across the 20-year period, independently of the distribution of the prediction values. The resulting trend maps summarize whether activity has intensified, declined, or remained stable at specific times of the day and seasons for each species.

For *Ae. albopictus*, the Mann-Kendall analysis (Fig. 4a) revealed a generalised trend throrough the year of increased activity in *Ae. albopictus* during the past two decades . However, the strongest and most consistent increase trends for this species occurred during the most climate-suitable months (June-October). For *Cx. pipiens*, the Mann-Kendall analysis (Fig. 4b) revealed a more heterogeneous temporal pattern of activity. A generalised trend of increased activity was identified throughout the year, with the clear exception of August (and some other months at specific time windows) where the activity significantly decreased according to model predictions.

## Discussion

Most organisms possess an internal circadian clock that synchronizes with environmental cues, primarily light and temperature. In mosquitoes, this circadian clock evolved to display time-dependent rhythms that regulate critical behaviors affecting their vectorial capacity, such as olfaction, immunity, host-seeking, blood-feeding, and ovipostion^15^. Consequently, understanding mosquito activity, particularly in densely populated urban areas, is critical for assessing human exposure risk to bites and optimising interventions to reduce nuisance and disease transmission^16^.

Here, we leveraged next-generation vector surveillance tools deployed across a Mediterranean city within the broader framework of a smart city approach, enabling real-time integration of entomological and environmental data into urban health monitoring systems. This allowed us to characterise the host-seeking activity patterns of two of Europe’s most epidemiologically relevant mosquito species: *Ae. albopictus* and *Cx. pipiens*^2^. Our unique entomological dataset, spanning temporal scales from hours to years, and its combination with high-resolution meteorological data, constitute a valuable asset for epidemiological surveillance in an urban context, providing unprecedented insight into how environmental factors shape mosquito activity from subdaily to seasonal scales and supporting data-driven public health interventions.

The present analysis relies on automated mosquito classification provided by the smart-trap system, whose performance has been extensively evaluated in previous studies under laboratory and field conditions^34–37^. In the field, the system achieves high balanced accuracy for discriminating mosquitoes from other non-Culicidae insects (>96%) and for genus-level classification between *Aedes* and *Culex* (>94%). Regarding sex classification, the balanced accuracy reported in two independent studies conducted in Spain and Italy ranged from   91–99% for *Ae. albopictus *females and   63–94% for *Ae. albopictus* males, while corresponding values for *Cx. pipiens* ranged from   88–99% for females and   63–83% for males^35,36^, highlighting both genus- and sex-specific differences as well as variability across experimental settings . Since the smart-trap classification algorithm was trained within a temperature range of 18–28 $^{\circ }$C, to minimize the impact of temperature-related misclassifications, we therefore restricted our analysis to the May–October period, which corresponds to the main mosquito season and largely falls within the validated thermal range of the sensor. Residual misclassifications are nonetheless expected to occur within this period. However, such errors are assumed to be randomly distributed in time and unlikely to produce systematic biases in the inferred diel patterns. Moreover, because the activity curves are derived from large numbers of detections aggregated across months and multiple years, classification uncertainty is expected to be residual and random rather than generate shifts in activity peaks. Given that the technology has already been validated in dedicated studies under appropriate temporal scales and analogous field conditions, and that the ASPB relies on these smart traps for operational use in combination with routine monthly surveillance, the objective of this study was not to revalidate the sensor. Rather, our aim was to move beyond validation and exploit the smart-trap data to address ecological and epidemiological questions.

Within this context, our results confirmed the presence of a bimodal activity pattern in both *Ae. albopictus* and *Cx. pipiens*, with two daily peaks tightly aligned with twilight transitions, coinciding with, or shortly after, sunrise and sunset events. *Culex pipiens* was more crepuscular and showed activity beyond dusk, extending into the scotophase. This species’ nocturnal endophilic behaviour could explain the low night-time activity detected by the smart traps, likely a consequence of outdoor sampling. In contrast, *Ae. albopictus* maintained a low but consistent activity between the peaks, with no complete inactivity even during full daylight hours. These findings are in concordance with earlier laboratory and field observations of mosquito diel behaviour for both species^15,38–40^; and highlight the importance of light cues in shaping mosquito circadian rhythms^15^.

The high temporal resolution offered by the smart-traps enabled a refined assessment of subtle inter-specific and sex-specific differences among mosquito field samples. While the morning activity peak was temporally aligned between species, the crepuscular peak started earlier (late afternoon) in *Ae. albopictus*, compared to the more strict crepuscular-nocturnal peak in *Cx.pipiens*. We also observed that male mosquitoes began activity slightly earlier than females during crepuscular peaks, whereas in morning peaks, both sexes were synchronized. This anticipatory behaviour of males in the crepuscular peaks could be associated with mating strategies, in which males arrive early and position themselves at swarm sites in anticipation of the arrival of females, as described for other *Culicidae* species^41^. Behavioural observations of male activity peaks in the field during mating may serve to optimize sterile insect technique operations and improve the success of *Aedes* invasive mosquito control^42^.

The effect of seasonality on daily mosquito activity was present for both targeted species. To avoid thermal stress and desiccation during warmer months, mosquitoes may adapt their activity to daytime periods with more favorable conditions. From cooler to warmer months, the morning peak’s intensity increased, but its timing remained stable. In *Ae. albopictus*, midday activity decreased and the crepuscular–nocturnal activity peak started later in warmer months. In *Cx. pipiens* the location of the crepuscular–nocturnal peaks shifted seasonally, becoming more nightly in warmer months. This behavioral plasticity reflects an adaptive response to seasonal climatic variation, highlighting the importance of temperature and photoperiod, as the main drivers of mosquito activity^26^.

Our RF model analysis identified photoperiod-related variables—specifically, the distances to sunrise and sunset—as key structural drivers of female activity patterns in both *Ae. albopictus* and *Cx. pipiens*. These variables were strong predictors for *Ae. albopictus* activity, whereas temperature emerged as the strongest predictor in *Cx. pipiens*. To interpret these patterns, we classified environmental drivers (*Zeitgebers*) into two functional roles: activators/suppressors, which act as “on/off switches” initiating or terminating activity within the circadian cycle (e.g., abrupt light transitions at dawn or dusk); and modulators, which fine-tune the amplitude, timing, or duration of activity without directly triggering its start or end (e.g., gradual temperature or humidity changes). In the RF framework, we interpret node purity (which reflects a variable’s capacity to create homogeneous splits) as an indicator of relevant activators or suppressors, since it captures their role in structuring activity peaks. In contrast, prediction accuracy (which reflects the model’s ability to reproduce observed patterns) better represents modulators, as it captures variables that explain the observed variability.

For *Ae. albopictus*, light-based *Zeitgebers* ranked highest for both node purity and prediction accuracy, indicating that they function as both activators and modulators of host-seeking behavior. Consistent with experimental findings^26,43^, abrupt light transitions at dawn and dusk serve as primary cues triggering daily activity peaks. Temperature also influences the seasonal onset and cessation of host-seeking activity^44^; however, in *Ae. albopictus* from temperate regions, temperature acted mainly as an activator/suppressor rather than a modulator, with light-based cues largely constraining the observed variation in activity. This supports the idea that, within tolerable thermal ranges (temperature activator “on”), *Ae. albopictus* host-seeking activity is primarily limited by light-related factors.

For *Cx. pipiens*, a primarily crepuscular and nocturnal blood-feeder, light-based factors and temperature also acted as key activators, as reflected by their high node purity scores. However, temperature (rather than light) emerged as its primary modulator, contributing most to prediction accuracy. The influence of temperature on the life-history traits of *Cx. pipiens* populations has been experimentally demonstrated, revealing the species’ sensitivity to the non-linear effects of temperature^19^. This supports our interpretation that, once darkness falls (light/dark activator “on”), nocturnal activity is constrained by thermal conditions. The observed role of temperature in shaping both seasonal and nightly variations in peak activity amplitude is consistent with our classification of temperature as a modulator.

In both species, rainfall acted as a secondary modulator, reducing activity when it occurred within the preceding 12 hours. This inhibitory short-term effect contrasts with the well-documented positive association between rainfall and mosquito population growth at seasonal and monthly scales, driven by the increased availability of breeding sites^45,46^. At finer temporal scales, however, rainfall appears to suppress host-seeking activity, as mosquitoes likely reduce movement or seek shelter during precipitation events. The scale-dependent influence of environmental predictors on behavioral traits underscores the importance of capturing mosquito behavior across a broad range of temporal scales, from hours to years.

Our findings indicate that the same environmental variable can play different regulatory roles in circadian rhythms depending on the species and the behavioural scales considered. The discrepancies between predictive accuracy (modulator-sensitive) and node purity (activator-sensitive) in the RF models highlight the complex interplay between diel and seasonal drivers of mosquito activity. This complexity reflects the dual role of the circadian clock in enabling both daily adaptation to environmental changes and coordination of seasonal processes such as diapause^21,47–49^.

The activator–modulator framework may also have an evolutionary interpretation: activators/suppressors could represent the primary mechanisms that align endogenous rhythms with environmental cues, while modulators provide higher-order refinements to fine-tune activity within those windows. Under this view, temperature can act as either an activator/suppressor, if crossing physiological thresholds that enable/suppress activity, or as a modulator when operating within the permissive range. With this perspective, the fact that temperature acts as main modulator in *Cx. pipiens* but not in *Ae. albopictus* may reflect long-term adaptations of the two species to their native climate regions.

As a temperate-climate and crepuscular-nocturnal species, *Cx. pipiens* is highly exposed to night-time temperature fluctuations that can shorten cool nights or prolong warm ones, making it strongly temperature-responsive, with temperature acting as a key seasonal and nightly modulator. In contrast, *Ae. albopictus*, a diurnal species native to tropical and subtropical climates, seems adapted to track seasonal photoperiod changes and rely on light (not temperature) to concentrate host-seeking behavior into dawn and dusk peaks. This timing overlaps with periods of increased outdoor human-host activity, enhancing the likelihood of host–vector contact^50^, and in tropical regions, it also helps avoid midday thermal and desiccation stress. At European latitudes, avoiding midday host-seeking activity due to thermal stress may not be necessary, but climate change could alter this pattern. Similarly, the seasonal interruption of activity through diapause, which facilitates colonization of temperate regions, may become less functional in some European areas under changing climatic conditions.

Backward forecasting over a two-decade period revealed significant temporal shifts in predicted female mosquito activity for both mosquito species. For *Ae. albopictus*, the temporal trend suggests a generalized intensification of daily and seasonal activity windows. This tendency of increased activity throughout the year, noteworthy during summer months, may reflect a progressive consolidation of favorable conditions for *Ae. albopictus* proliferation, such as higher temperatures, which is aligned with the thermophilic nature of this species^20^. The observed increase in autumn and winter activity is also in concordance with previous empirical evidence of diapause inhibition and extended activity windows that match anomalous high temperatures during winter months^44^. For *Cx. pipiens*, predicted activity generally increased throughout the year, except in August (and in certain specific periods, mostly around sunset during the winter months) when activity decreased. This August activity decline is likely due to extreme temperatures during summer and the recent rise in heatwave frequency^51^, which may suppress host-seeking activity as mosquitoes avoid desiccation and seek refuge. The reduced activity in winter around sunset may reflect an interaction between light-based activators and low-temperature modulators, shifting or dampening activity peaks. Alternatively, this decrease might reflect an enhanced ability of *Cx. pipiens* to seek and bite hosts indoors, thereby partially buffering against adverse outdoor conditions.

Although the RF model was trained on smart-trap observations collected between May and October (the period whose temperature range matches the conditions used to train and validate the sensor’s automated classification), its environmental predictors remain defined throughout the entire year, allowing the model to be applied beyond the training window. Nonetheless, we acknowledge that this approach implicitly assumes that the climate–activity relationships observed between 2021 and 2024 remained stable throughout the 2004–2024 period. In practice, mosquito activity may also be shaped by processes and non-climatic drivers that are not explicitly represented in the model. These may include: the host availability and behavior^52,53^, the interaction between host cues and the vector’s inner circadian clock^54^, the vector indoor host-seeking behaviour^55^, the micro-habitat environmental conditions^52,56^, the vector’s nutritional status^57^, the implementation of control measures^58^, the presence of artificial light at night^59,60^, etc. These factors may be responsible, at least in part, of the moderate/low explanatory power of the model (R²= 0.53 and R$^{2}$= 0.33 for *Ae. albopictus* and *Cx. pipiens* respectively). This suggests that model predictions, specially those generated outside the warm season, should be interpreted with appropriate care. Even so, the year-round projections remain a meaningful approximation, based on the available environmental predictors, for visualising environmentally driven temporal trends.

The identification of potential shifts in mosquito activity patterns, driven by key environmental drivers with distinct activator and modulator roles, underscores the need to revisit traditional assumptions underlying vector control. Artificial light at night and anomalously high temperatures late in the mosquito season have been shown to disrupt the natural rhythms of medically important mosquito vectors, respectively altering biting behavior^59,61^ and diapause initiation^62^. These disruptions may not only increase human–mosquito contact and the risk of MBD transmission but also reduce the effectiveness of static control calendars. Access to real-time mosquito activity data is therefore critical for optimizing adulticide interventions during outbreaks, where precise timing can drastically improve control outcomes^63^. Also, dynamic mosquito activity calendars should be taken into account not only when designing intervention strategies, but also in public information, awareness, and education programs.

The integration of smart traps into urban vector surveillance networks offers a powerful means to capture mosquito activity across diel, seasonal, and interannual scales, providing the temporal resolution needed to identify true spatiotemporal risk windows. By combining species-specific activator–modulator dynamics with real-time IoT-enabled data streams, public health agencies can move from static, calendar-based interventions to adaptive, evidence-driven vector control. This approach enhances the ability to anticipate and respond to changes in mosquito behaviour driven by environmental variability, climatic anomalies, or anthropogenic factors.

While our network provided high temporal resolution, its spatial coverage was relatively limited, and local variability in microclimatic conditions around trap sites may have contributed to subtle differences in observed activity patterns. Given the nature of the available data and the configuration of the smart-trap network (designed for manageable routine surveillance) this study primarily addresses temporal rather than spatial dynamics of mosquito activity. Accordingly, fine-scale spatial features known to influence mosquito activity, such as land use or urban structure, were not included, as they do not vary at an hourly resolution and therefore cannot be meaningfully linked to within-day changes in activity. In the short term, efforts should focus on optimizing trap density, placement, and operational schedules to enhance outbreak responsiveness and control efficiency. Also, expanding these scalable networks beyond Mediterranean cities into temperate and tropical regions would be desirable, not only to enable comparative studies of mosquito chronobiology but also deepen our understanding of how environmental drivers shape vector activity across diverse ecological and climatic contexts, ultimately strengthening early-warning capacity and supporting precision public health interventions against MBD.

## Methods

### Data gathering

**Fig. 5:**
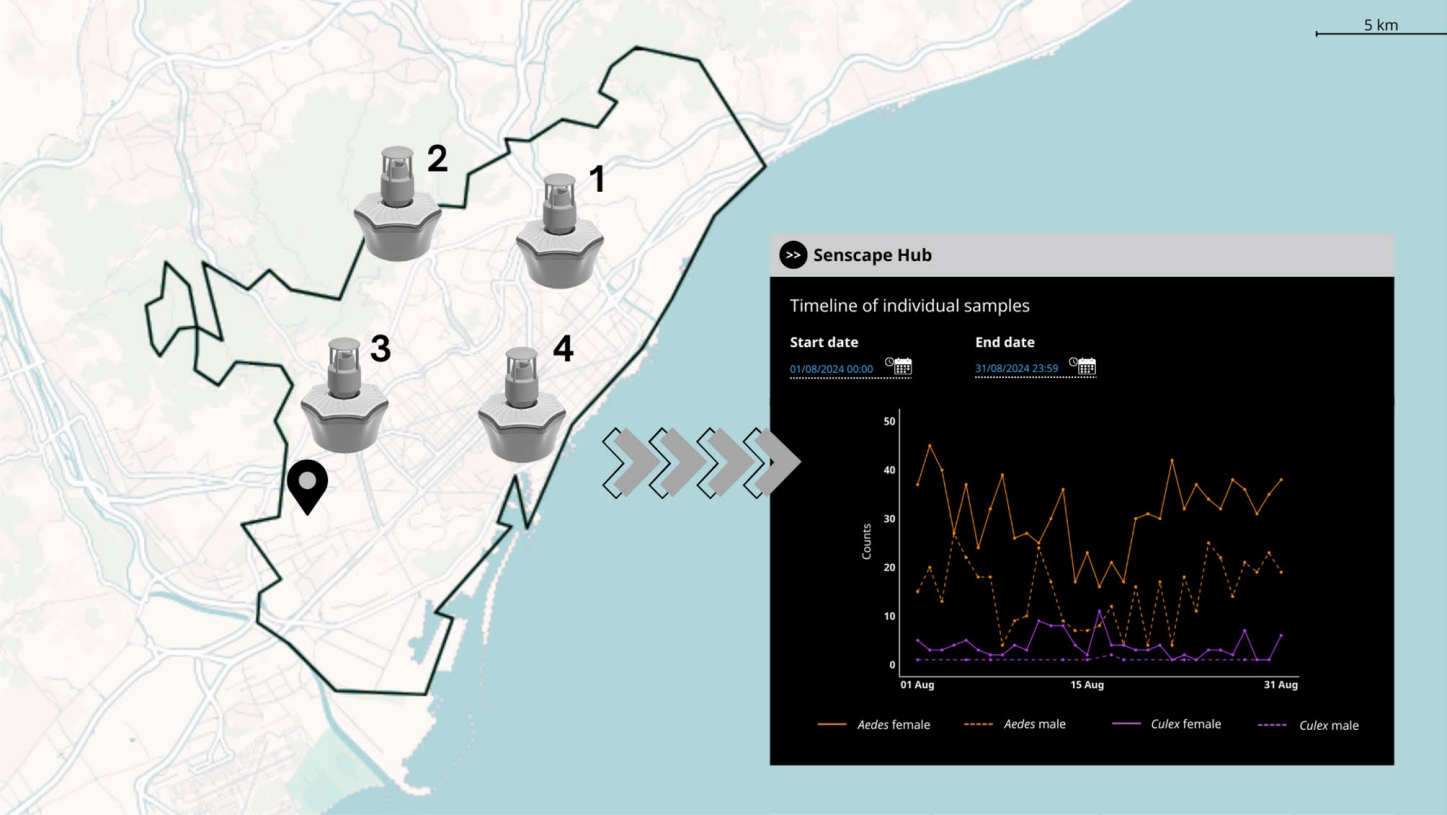
Graphical composition showing the metropolitan area of Barcelona (Catalunya, NE Spain) with the location of the different smart-traps (1–4) and the meteorological station (geolocation icon); and an example of the smart-trap output from the Sencape Hub web portal for a daily temporal scale. The map was generated using R version 4.5.1 (https://www.r-project.org). Administrative boundary data were obtained using the “mapSpain” package^64^.

Four smart-traps were installed by the Public Health Agency of Barcelona in the metropolitan area of Barcelona (Catalunya, NE Spain), operative from 2021 to 2024 (Fig. 5): Trap 1 - St Andreu (41$^{\circ }$25’59.3”N 2$^{\circ }$11’27.9”E); Trap 2 - Horta (41$^{\circ }$26’21.2“N 2$^{\circ }$08’49.8”E); Trap 3 - Pedralbes (41$^{\circ }$23’19.6“N 2$^{\circ }$07’01.2”E); Trap 4 - Zoo (41$^{\circ }$23’10.8“N 2$^{\circ }$11’22.9”E). The traps were manually inspected monthly by expert technicians as part of their routine vector surveillance program. Mosquitoes present in the catch bags inside the traps were identified at species level. Three different mosquito species were present in the samples: *Ae. albopictus*, *Cx. pipiens* and *Culiseta longiareolata*. Hence, mosquitoes classified by the smart-trap as *Aedes* or *Culex* were necessarily from the species *Ae. albopictus* and *Cx. pipiens*, respectively. When a *Cs. longiareolata* entered the trap, the smart-trap classified it as a non-target insect. See González-Pérez et al. (2024) for more information about the field validation of the smart-trap classification results^35^.

Automated real-time data obtained from the smart-traps (see representative example in Fig. 5) in the period comprised between the 1st of January 2021 to the 31st of December 2024 was downloaded from the Senscape Hub portal (https://www.senscape.eu) of Irideon S.L, via API request in a .cvs file. We downloaded two datasets: the first one contained real-time information about mosquito captures (the genus and sex of each mosquito recorded, the number and location of the trap, and a series of labels associated with the recording event such as date, time, temperature, and relative humidity); the second dataset contained information, generated each 30 min, on whether a smart-trap was active or not at that time, and when active, reported temperature and humidity values.

More extensive information on environmental data was downloaded from the net of automated weather stations of Barcelona (XEMA) via API request to the Meteorological Service of Catalunya (Meteocat) for the same period. The meteorological station named Barcelona-Zona Universitària [X8] (41$^{\circ }$22’45.1“N 2$^{\circ }$06’19.4”E) (Fig. 5) was selected among the four stations available in the city for being the most representative of the weather of Barcelona, according to the expertise of the Public Health Agency of Barcelona. Weather data was downloaded in a .csv file and contained information about temperature, relative humidity, total solar radiation, and precipitation. The temporal resolution of weather data was 30 min.

### Daily and seasonal patterns of mosquito activity

Raw mosquito data collected by the smart-traps and downloaded from the Senscape Hub web portal was used to represent the daily activity peaks of *Ae. albopictus* and *Cx. pipiens* using the “Activity” package in R^65^. As date and time have a circular nature, the estimation of the activity level was done by fitting a flexible circular kernel distribution and calculating the overall proportion of time active from this, following the approach of Rowcliffe et al.^66^. To standardize the outputs related to mosquito activity across latitudes and seasons, local time was transformed by the double anchoring method described in Vazquez et al.^67^, attuning it to sunrise and sunset events.

Only data comprised between May and October covering mosquito season was included (see Supplementary Note S1 and Supplementary Fig. S6 for further information). The activity of both targeted species was represented for female and male mosquitoes; and across different seasons.

### Modeling female mosquito host-seeking activity

Since mosquito activity peaks are expected to occur only under specific environmental conditions, such as particular time windows relative to sunrise and sunset and/or optimal temperature ranges, we used a set of explanatory environmental variables to model the temporal variation in hourly female mosquito abundance. Hourly female mosquito abundance, as inferred from the smart-trap counts, was interpreted as a proxy for mosquito host-seeking activity, based on the assumption that, at this fine temporal scale, higher mosquito counts per hour reflect increased activity of that species during that period. Data independence was assumed, as each detection corresponded to a unique specimen, which was immediately trapped in the catch bag after triggering the sensor.

Two modeling approaches were considered: (a) aggregating all female mosquito counts from different traps by hour and standardizing them by sampling effort (i.e., the number of active traps per hour), using hourly weather data from the XEMA station; or (b) modeling hourly mosquito counts separately for each trap, using weather data recorded directly by each smart-trap during detection events. The former approach omits spatial variation but gains statistical power through data aggregation, whereas the latter accounts for spatial heterogeneity but involves more noise due to modeling individual trap data. After consideringboth approaches, we determined that the first option was more appropriate for several reasons. First, the daily activity patterns inferred independently from each smart-trap were highly similar (see Supplementary Fig. S7), indicating that separate trap-level modelling does not provide additional insight into the temporal structure of mosquito activity. Second, the limited number of smart-traps available prevents a robust analysis of micro-scale spatial heterogeneity, making aggregation a more appropriate strategy for a city-scale activity analysis. Third, environmental data recorded by individual smart-traps occasionally contained missing values (see Supplementary Fig. S8), which further reduced the feasibility of trap-level modelling. Besides, temperature and humidity measurements recorded by the smart-traps and the XEMA station were highly correlated , suggesting that microscale environmental variation was not a major factor influencing mosquito activity in this context. Finally, aggregating trap-level counts reduced the zero-inflation, dataset size and processing time, resulting in a more manageable and computationally efficient modelling framework. Overall, the aggregated approach provides a clearer and more robust representation of temporal mosquito activity dynamics at the city scale.

Subsequently, to prepare the data for modeling, the total hourly counts of female *Ae. albopictus* and *Cx. pipiens* recorded by the four smart-traps were aggregated by date-time and standardized by sampling effort (SE), defined as the number of active traps at that specific time. The resulting response variables of the model were then the total number of female *Ae. albopictus* and *Cx. pipiens* mosquitoes per active trap per hour which were modeled separately.

As noted above, weather data was obtained from the Barcelona - Zona Universitària [X8] XEMA station. The set of explanatory variables considered for the analysis were: temperature (including average values of temperature for 6, 12 and 24 prior hours), relative humidity (including average values of humidity for 6, 12 and 24 prior hours), precipitation (including cumulative values of precipitation for 6, 12 and 24 prior hours), solar radiation, activity degree hours (ADH) (including cumulative values of ADH for 6, 12 and 24 prior hours), distance to sunrise and sunset events, day length, hour of the day (HOD) and day of the year (DOY). A complete list of the considered variables, with their names, description, and units is given in Supplementary Table S1. A detailed description of the ADH feature is provided in Supplementary Note S2 and Fig. S9. For the final dataset, only data collected between May and October covering mosquito season was included (see Supplementary Note S1 and Supplementary Fig. S6 for further information).

We computed Pearson’s correlation coefficients for each pair of explanatory variables (see Supplementary Fig. S10), and excluded variables with a correlation coefficient greater than 0.8 from further analysis. To assess potential multicollinearity among the remaining variables, we calculated the Variance Inflation Factor (VIF); variables with a VIF greater than 5 were also excluded. The final explanatory variables included in the analysis were: hourly temperature, average relative humidity during the last 12 hours, cumulated precipitation during the last 12 hours, distance to sunrise and distance to sunset.

We assessed the distribution of the response variables using histograms, Q–Q plots, and formal normality tests, which revealed non-normal, zero-inflated distribution patterns(Supplementary Fig. S11). Given the nature of the date, and the evident non-linear relationships between the explanatory and response variables (see Supplementary Fig. S12), we selected a Random Forest (RF) model. A RF is an ensemble machine-learning algorithm that builds multiple decision trees using bootstrap samples and random subsets of predictors, and combines their predictions to capture complex non-linear relationships without requiring distributional assumptions, making it well suited for zero-inflated, non-normally distributed ecological data. To build the RF model, the dataset was divided into training (70%) and test (30%) subsets. A 4-fold cross-validation was applied to the training set to optimize the hyperparameters, and the best-performing model was subsequently evaluated on the test set. Model performance was assessed using the root mean squared error (RMSE) and the coefficient of determination (R^2^).

To assess the contribution of each predictor variables to the RF model, we examined two variable-importance metrics: (i) the percentage increase in mean squared error (%IncMSE), which measures the rise in prediction error when a given variable is permuted and therefore reflects its influence on predictive accuracy; and (ii) the increase in node purity (IncNodePurity), which captures how much each variable improves the homogeneity of splits within the regression trees. A variable with high %IncMSE is essential for maintaining the global predictive performance of the model, indicating that it provides key temporal structure to mosquito activity patterns. In contrast, a variable with high IncNodePurity is particularly effective at generating homogeneous splits within the regression trees, meaning that it discriminates well between low- and high-activity conditions. Together, these metrics provide complementary insights into the role of each environmental driver in shaping hourly mosquito activity.

### Historical evolution of female mosquito activity in the city of Barcelona

We applied the RF model to generate retrospective predictions of female mosquito activity, enabling an assessment of how activity patterns have evolved in Barcelona over the past two decades. We downloaded hourly climate data in .nc files from Copernicus Climate Data Store (https://cds.climate.copernicus.eu) via API request, for a 1 km^2^ box area around the [X8] XEMA station (coordinates: 41.37919, 2.10540, 41.37819, 2.10640) for each year from 2004 to 2024. The downloaded variables were: air temperature (2m above the ground) in K, temperature at dew point in K, total precipitation in m; and surface solar radiation downwards in J/m^2^. Files were transformed to a .csv format and variable units were converted according to previous weather variable units used for modeling (see Supplementary Table 1). Relative humidity was calculated from air temperature, and temperature at dew point, by applying an improved version of Magnus formula^68^. The validated RF model was used to make retrospective predictions of hourly female mosquito activity . A non-parametric statistical test, i.e. Mann-Kendall test, was used to detect the presence of temporal trends within the time-series data of female mosquito activity using the “Kendall” package in R^69^.

## Supplementary Information

Supplementary Information.

## Data Availability

The datasets generated and analysed during the current study, as well as code used to carry out the analysis and graphs, are available at the [MOSQUITO_ACTIVITY_BCN] GitHub repository, [https://github.com/maigonpe/MOSQUITO_ACTIVITY_BCN].
