# Supplementary material for: Unravelling the activity rhythms of urban vector mosquitoes with smart-trap technology
Source: Sci Rep. 2026 Feb 14;16:9075. doi: 10.1038/s41598-026-38795-y (PMC12992545; doi:10.1038/s41598-026-38795-y)
Supplement: Supplementary file 1 — Supplementary Information. [file 41598_2026_38795_MOESM1_ESM.pdf]

Supplementary Information

Figure S1. Variance importance plot of the RF model for *Ae.albopictus* (**a**) and *Cx. pipiens* (**b**).

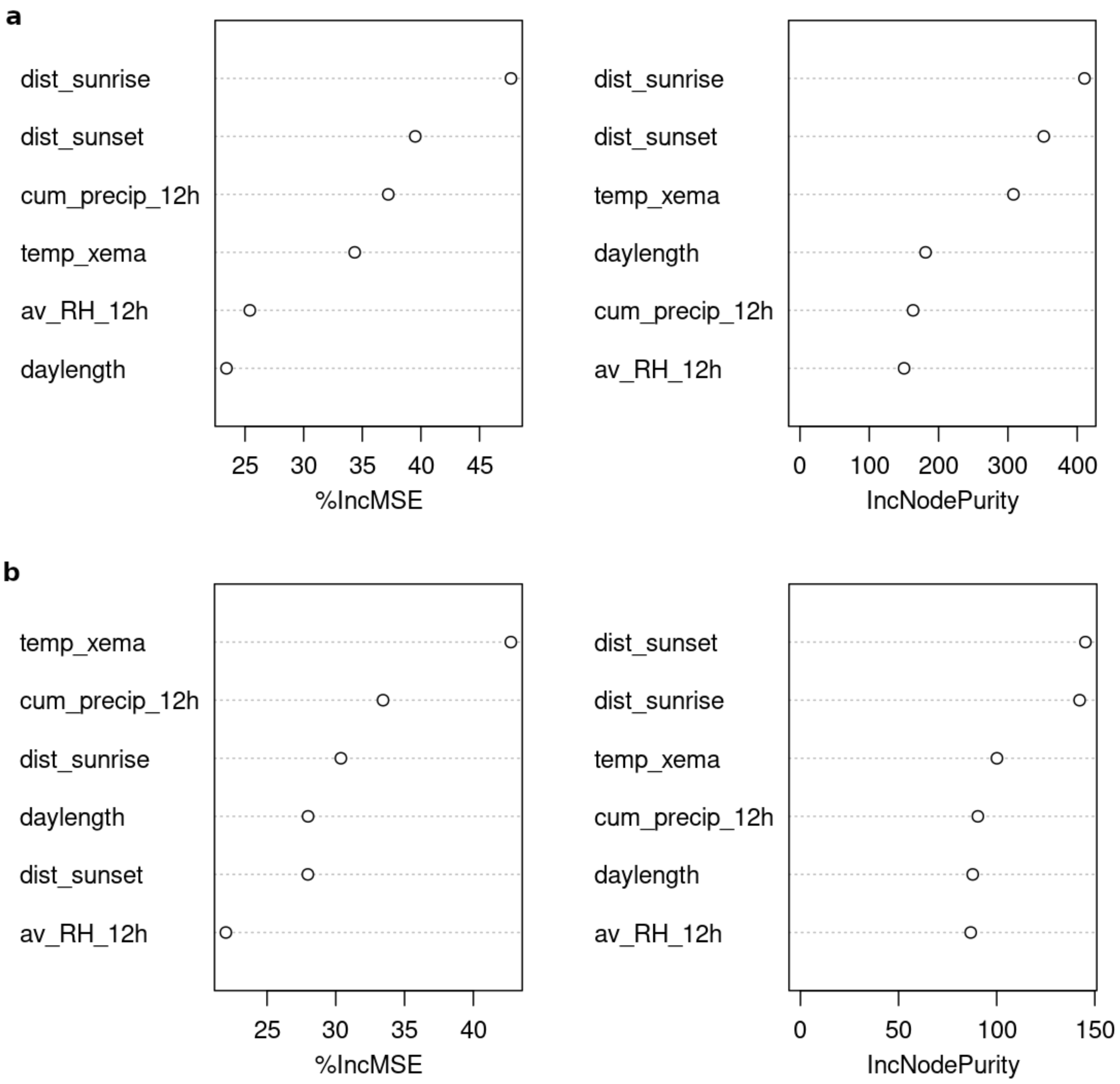

Figure S2. Seasonal evolution of temperature and photoperiod throughout the study period (2021-2024)

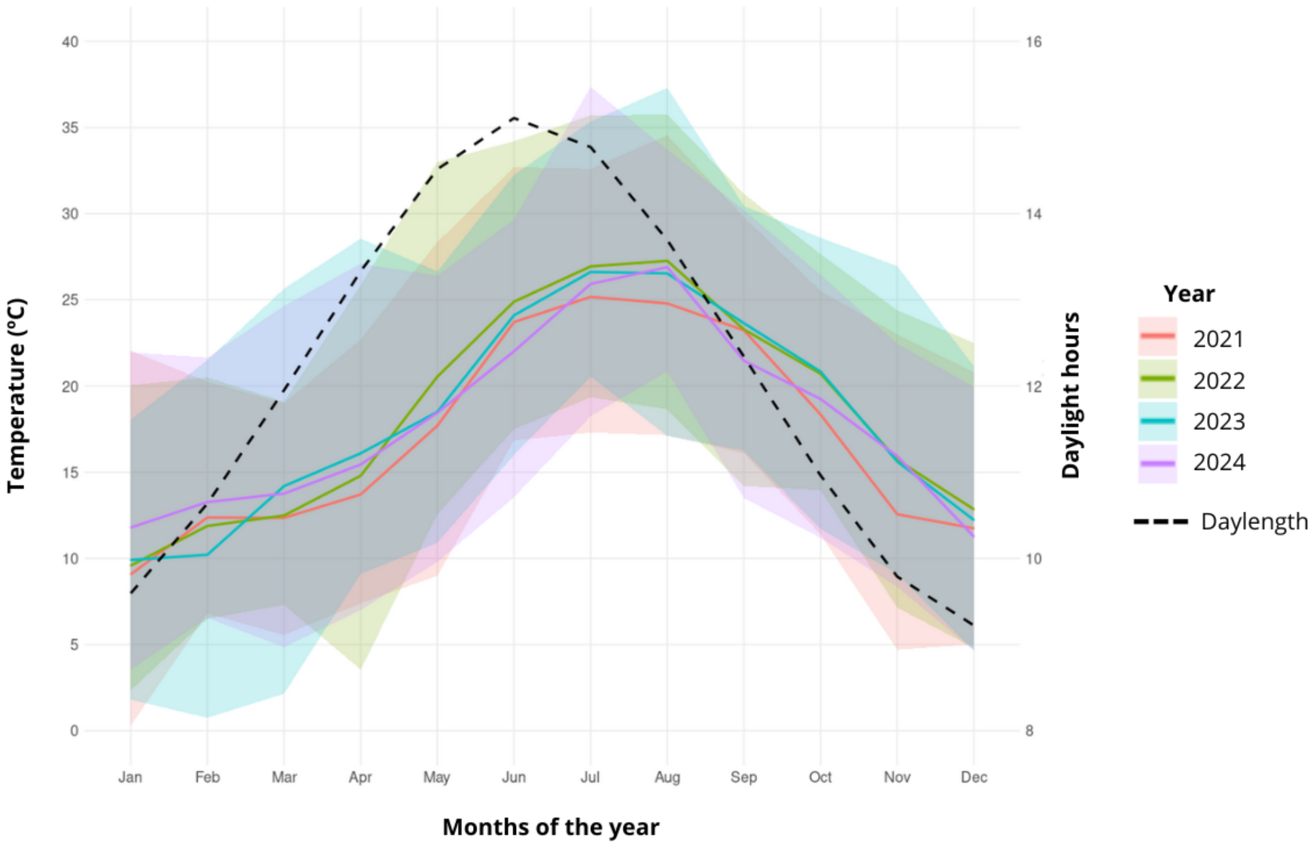

Figure S3. Historical evolution of predicted *Ae. albopictus* female activity from 2004 to 2024

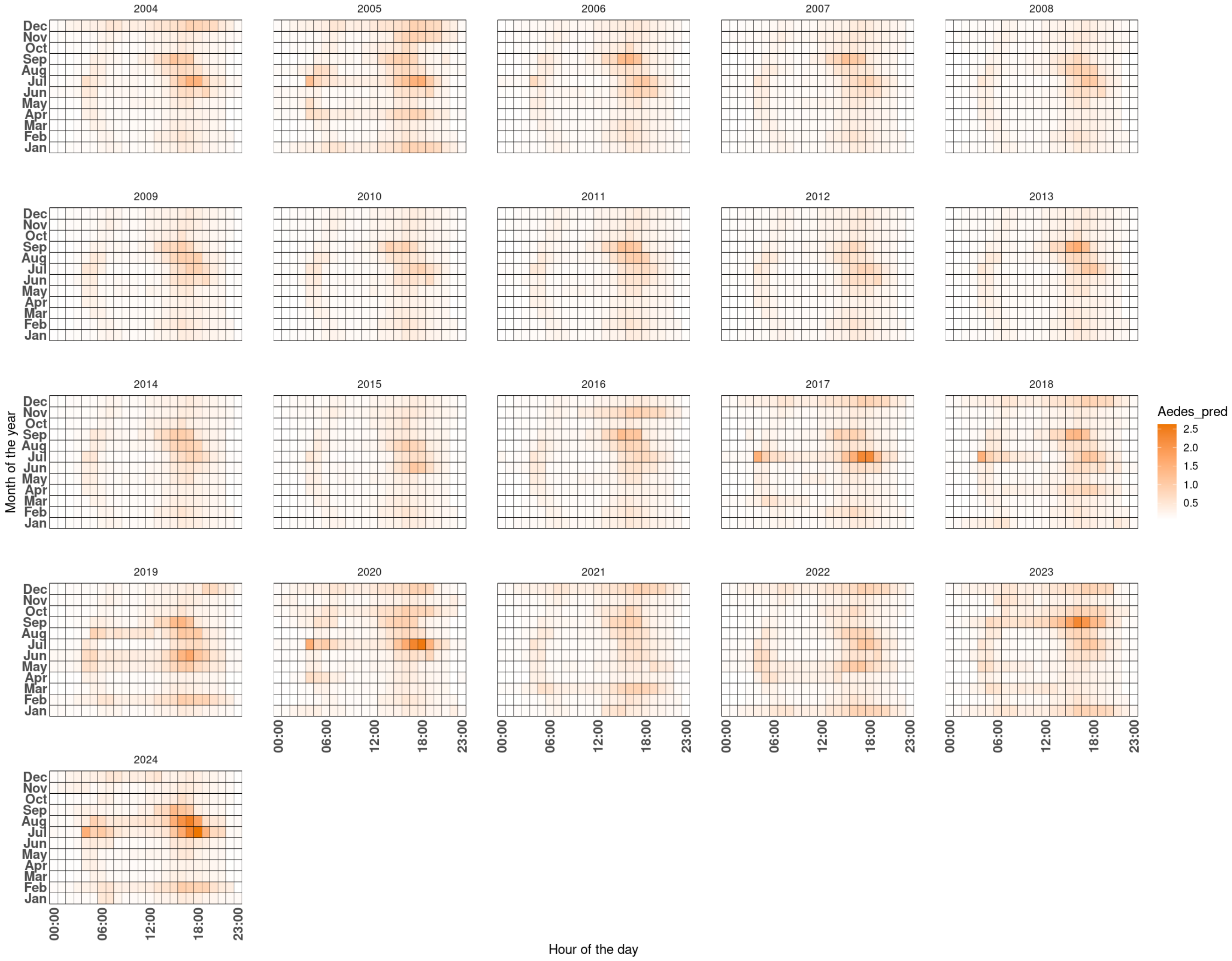

Figure S4. Historical evolution of predicted *Cx. pipiens* female activity from 2004 to 2024

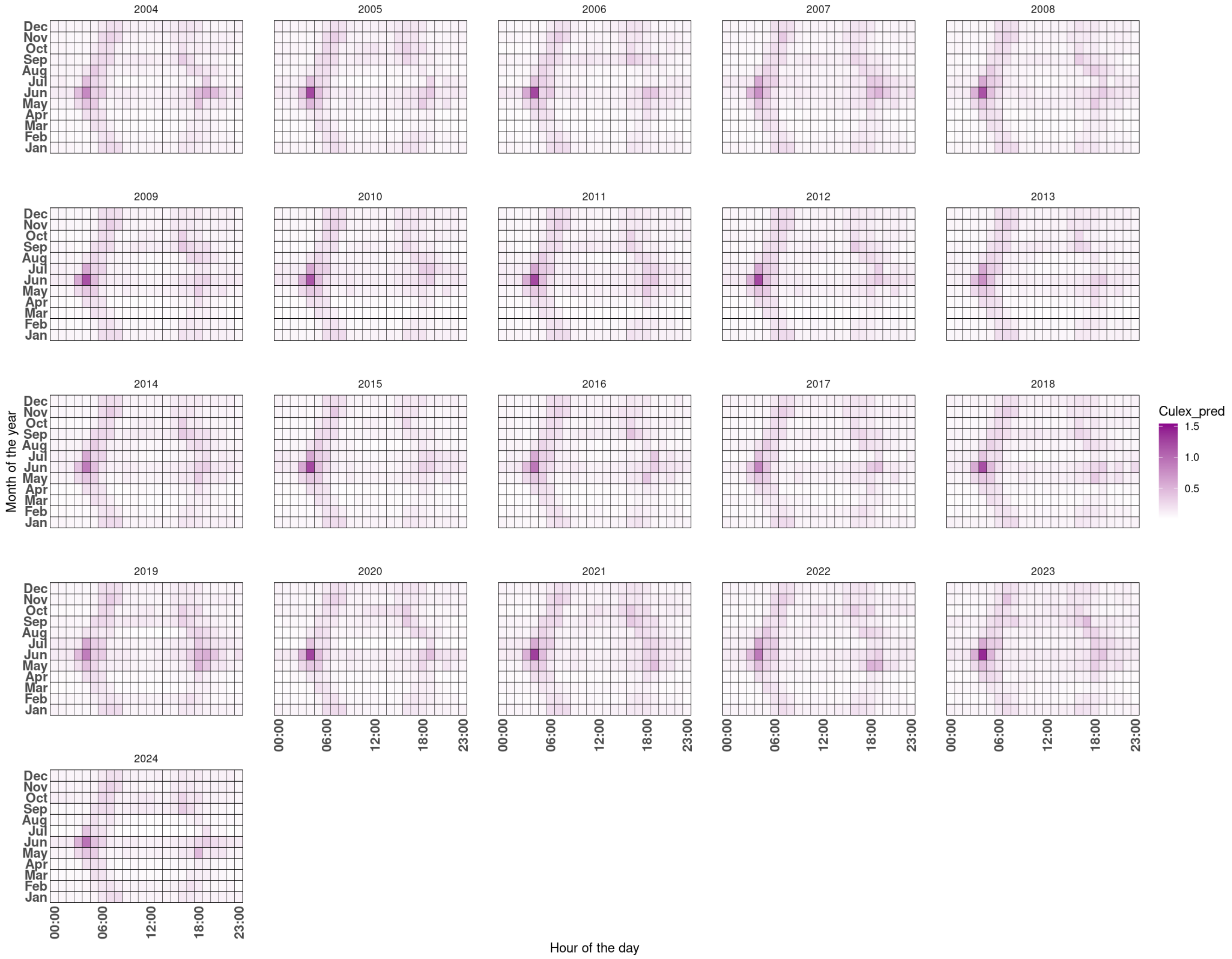

Figure S5. Comparison between observed and predicted activity in 2024 obtained from smart-trap data and RF model respectively, for the species *Ae.albopictus* (**a**) and *Cx. pipiens* (**b**).

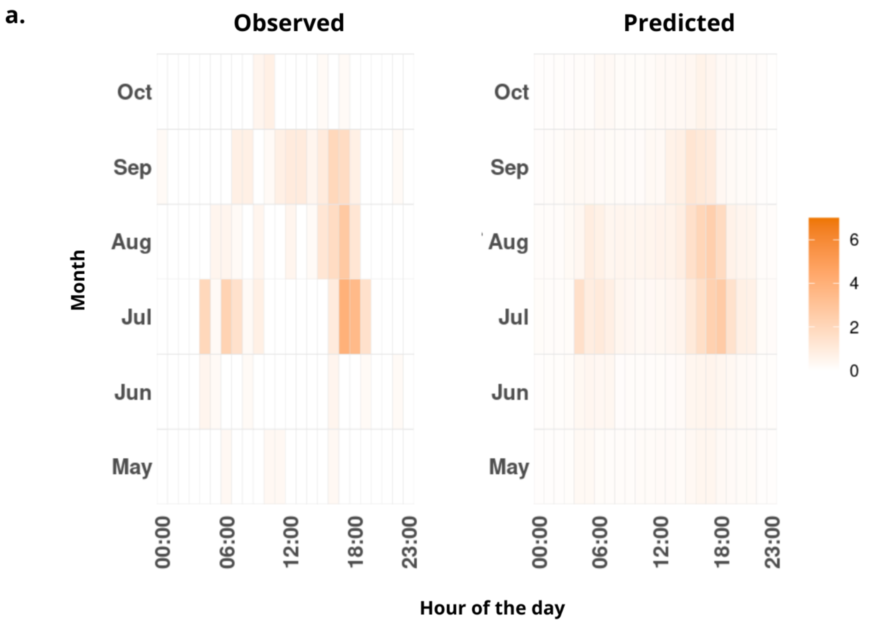

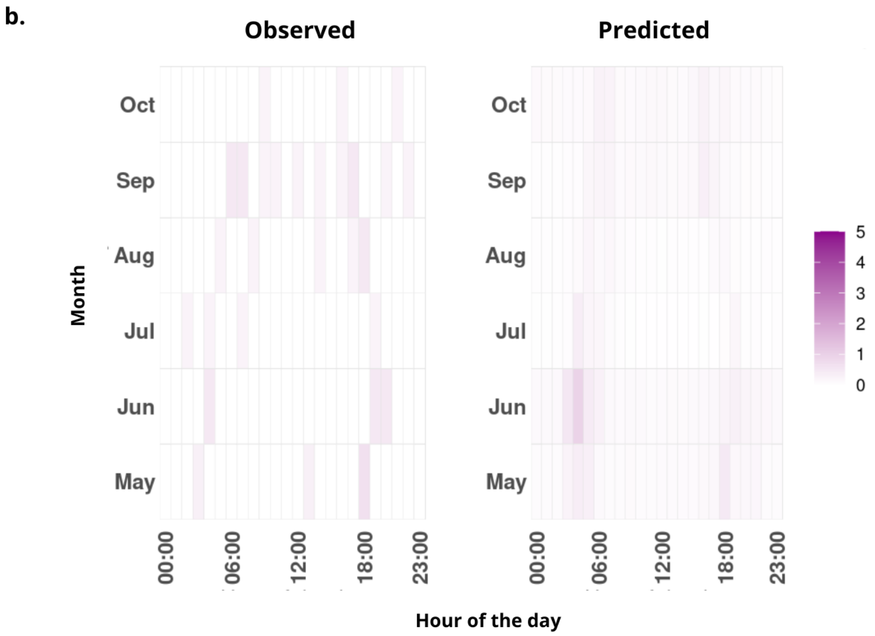

Note S1: Duration of the study period

According to González-Pérez et al. (2024)[5], the smart-trap sensor was trained to classify the genus and sex of *Ae. albopictus* and *Cx. pipiens* under three temperature regimes (18ºC, 23ºC and 28ºC). Below 18ºC or above 28ºC, the sensor might commit some misclassifications (it has not been studied yet). In the attempt to retrieve the maximum classification accuracy from the smart-traps, the authors of the present paper decided to be conservative and select for study period only these months where almost 50% of the days the average daily temperature (obtained from XEMA station) was between these thermal limits. As depicted in Fig. S6, the percentage of days within a month where the average temperature was above 28ºC was never below the 50% in the four years that lasted the study. On the other hand, only from May to October (both included) the percentage of days where the temperature was under 18ºC was below the 50% (with the exception of May 2021). This is why the study period was comprised between May and October.

Figure S6. Percentage of days within a month where the average temperature is below 18ºC or above 28ºC.

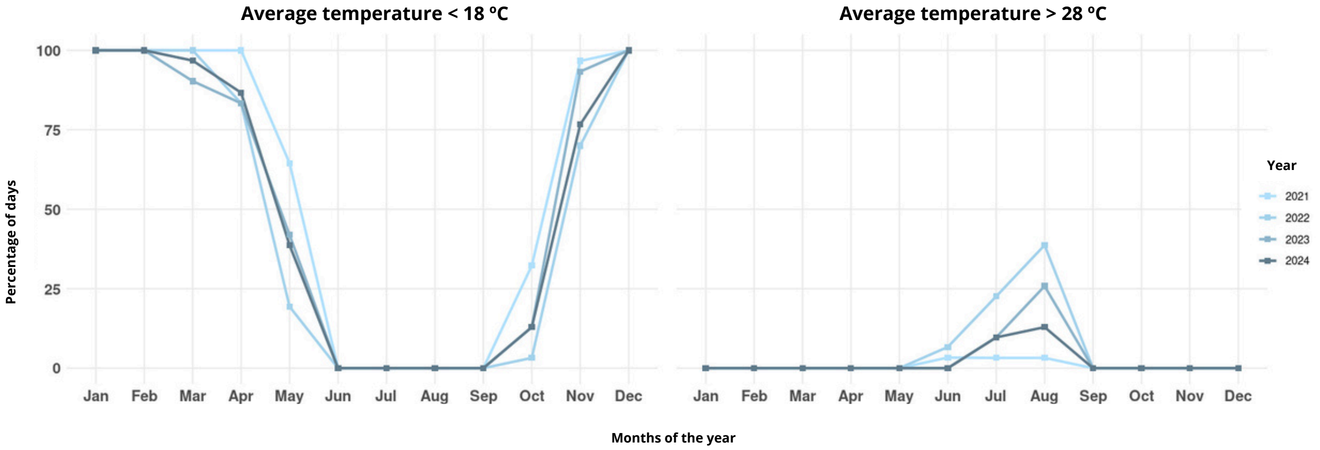

Figure S7. Diel mosquito activity patterns extracted from individual smart-traps

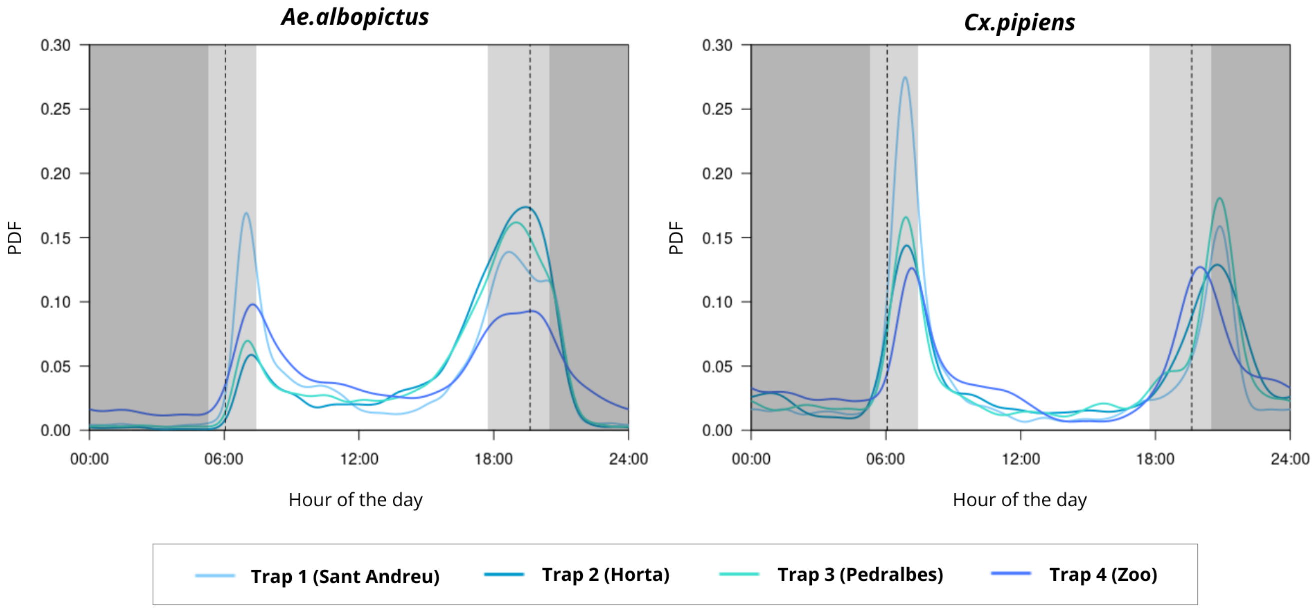

Figure S8. Comparison of temperature (**a**) and relative humidity (**b**) data obtained from XEMA station (light pink) and from the smart-traps (light blue) for the study period

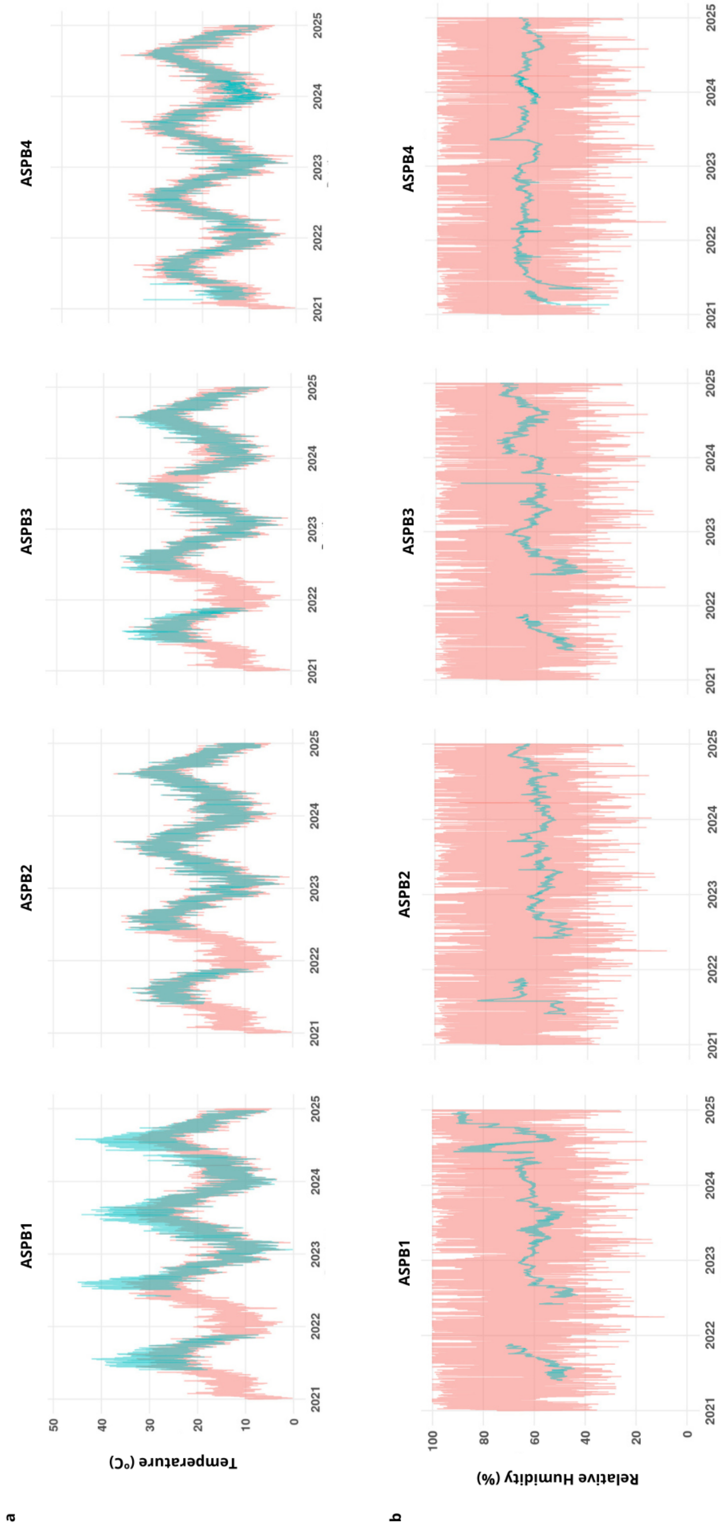

Note S2: Activity Degree Hours (ADH)

The Activity Degree Hours feature (ADH) has been conceived in the present paper as a linear temperature-driven model for insect activity. The underlying idea is that, as poikilotherm organisms, the activity of mosquitoes is constrained to a minimum and a maximum thermal limits. Hence, within optimum temperature ranges of activity, and as environmental temperature decreases, their rates of activity will also decrease and cease at the minimum temperature limit (base temperature). Within this same optimum range, and as environmental temperature rises, activity rates will increase up until a maximum temperature limit, above which activity will cease.

The concept of ADH is derived from the original average method proposed by Baskerville and Emin in 1968[1] to calculate the Growing Degree Days (GDD), which has been extensively applied to the fields of agronomy and entomology to optimize crop production and for pest management. The formula describing ADH was directly adapted from the formula described in Hachisuca et al. (2023)[2] for Growing Degree Hours (GDH), but in this case, incorporating a maximum thermal limit.

The mathematical function for ADH is defined as:

$$ADH(T) = \begin{cases} 0, & T \leq T_{\text{base}} \text{ o } T \geq T_{\max} \\ T - T_{\text{base}}, & T_{\text{base}} < T < T_{\max} \end{cases}$$

In the function, T = hourly temperature in ºC; Tbase = base temperature below which mosquito activity is not possible; and Tmax = maximum temperature above which mosquito activity is not possible. If T is below Tbase or above Tmax, ADH equals 0. If T is between Tbase and Tmax, then ADH = T - Tbase. Since the temporal resolution of the meteorological data obtained from XEMA (MeteoCat services) is 30 min, T was calculated as the average temperature per hour. The reference temperature values for Tbase (10ºC) and Tmax (35ºC) were extracted from the existing literature about thermal limits for the species of interest of this study *Cx. pipiens*[3] and *Ae. albopictus*[4]; and also from the analysis of our own data (see Supplementary Figure S9 for more details).

Figure S9. Thermal limits of *Aedes albopictus* and *Culex pipiens*. Temperature data gathered from XEMA stations when mosquitoes are present.

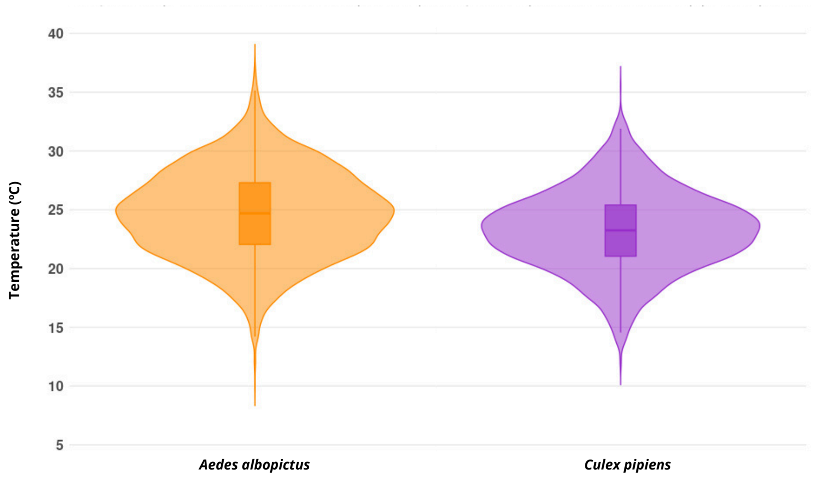

Figure S10. Correlation heatmap showing the correlation index between pairs of predictor variables.

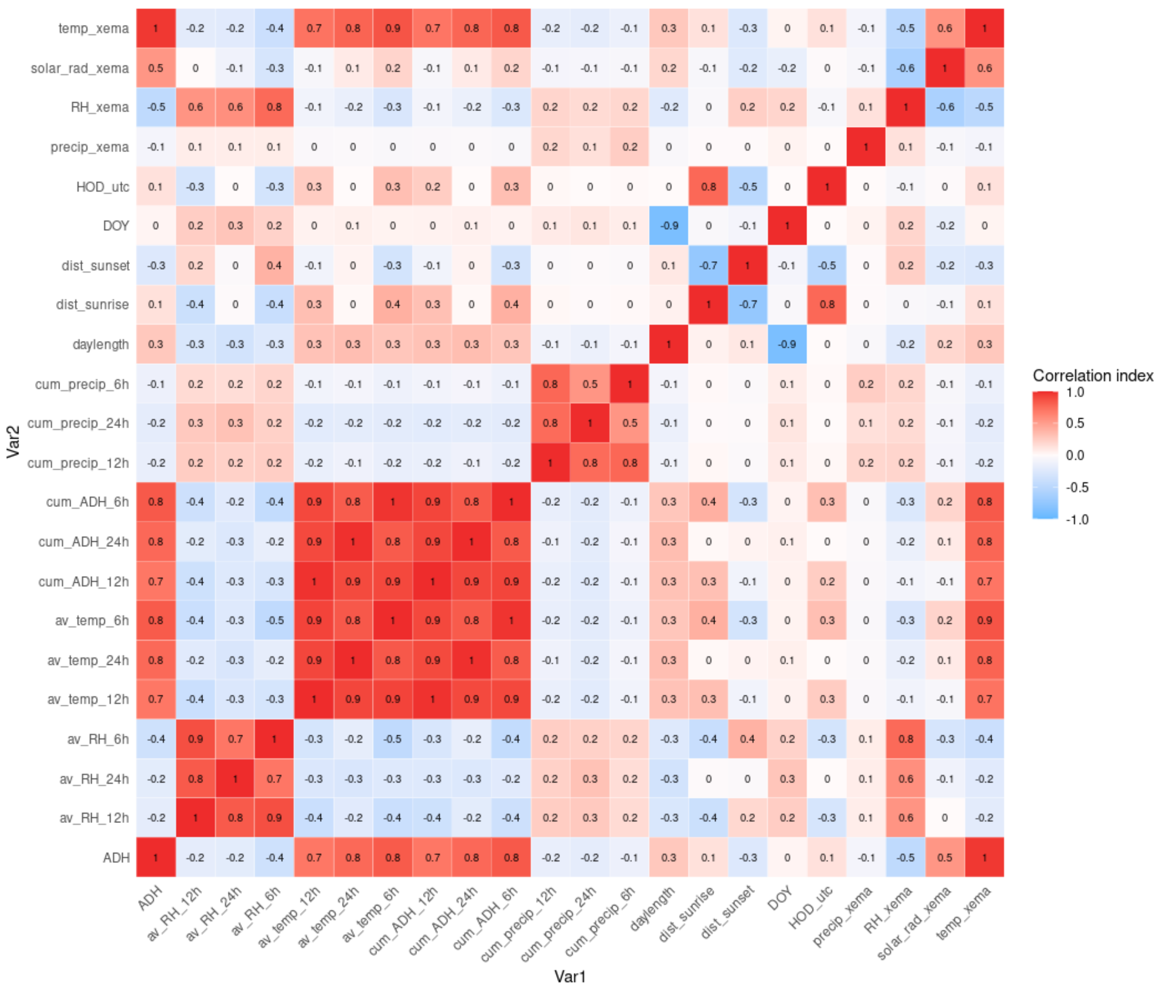

Figure S11. Data exploration: assessing data distribution with an histogram (**a**); and normality assumption with a Q-Q plot and an Anderson-Darling normality test (**b**).

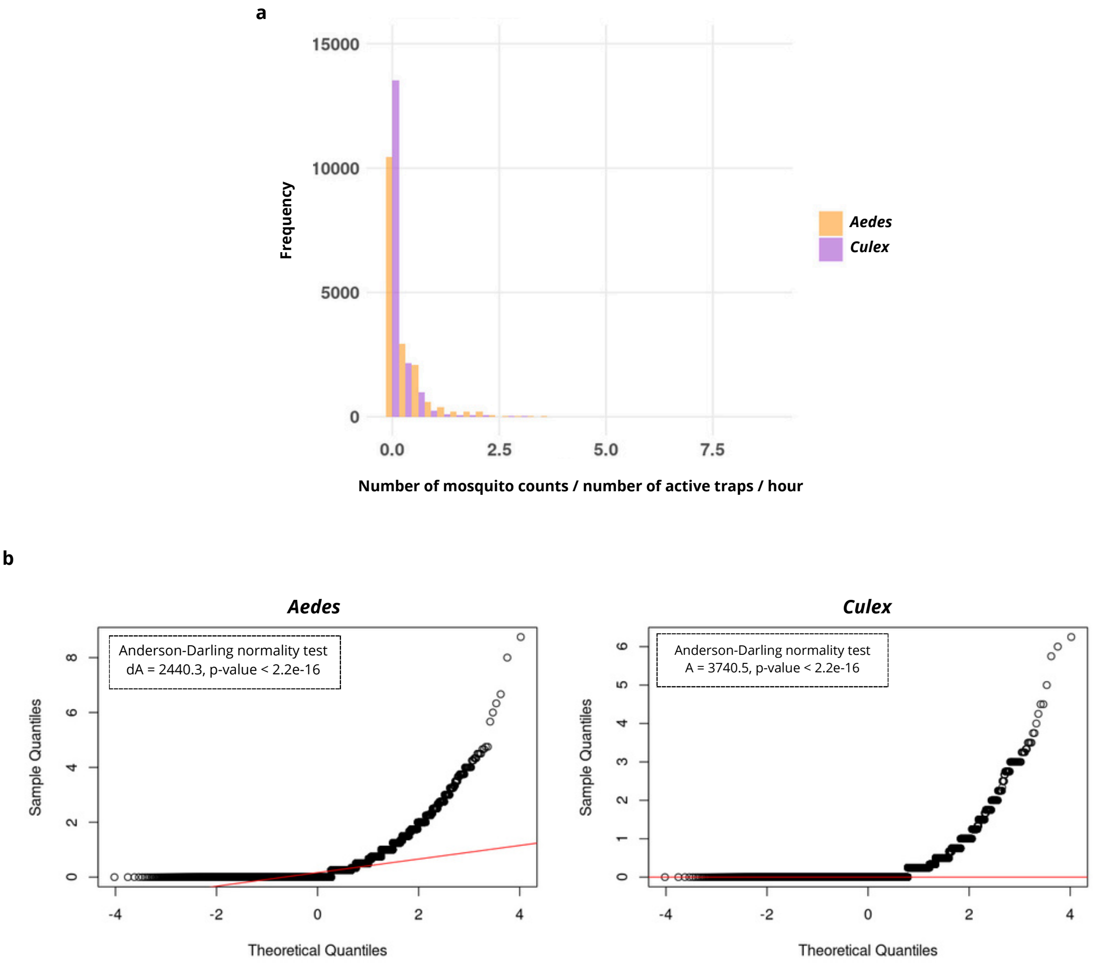

Figure S12. One-to-one relationships between the explanatory variables and the response variables (number of mosquito counts per number of active traps per hour). Non-linear relationships are deduced for *Ae.albopictus* (**a**) and *Cx. pipiens* (**b**).

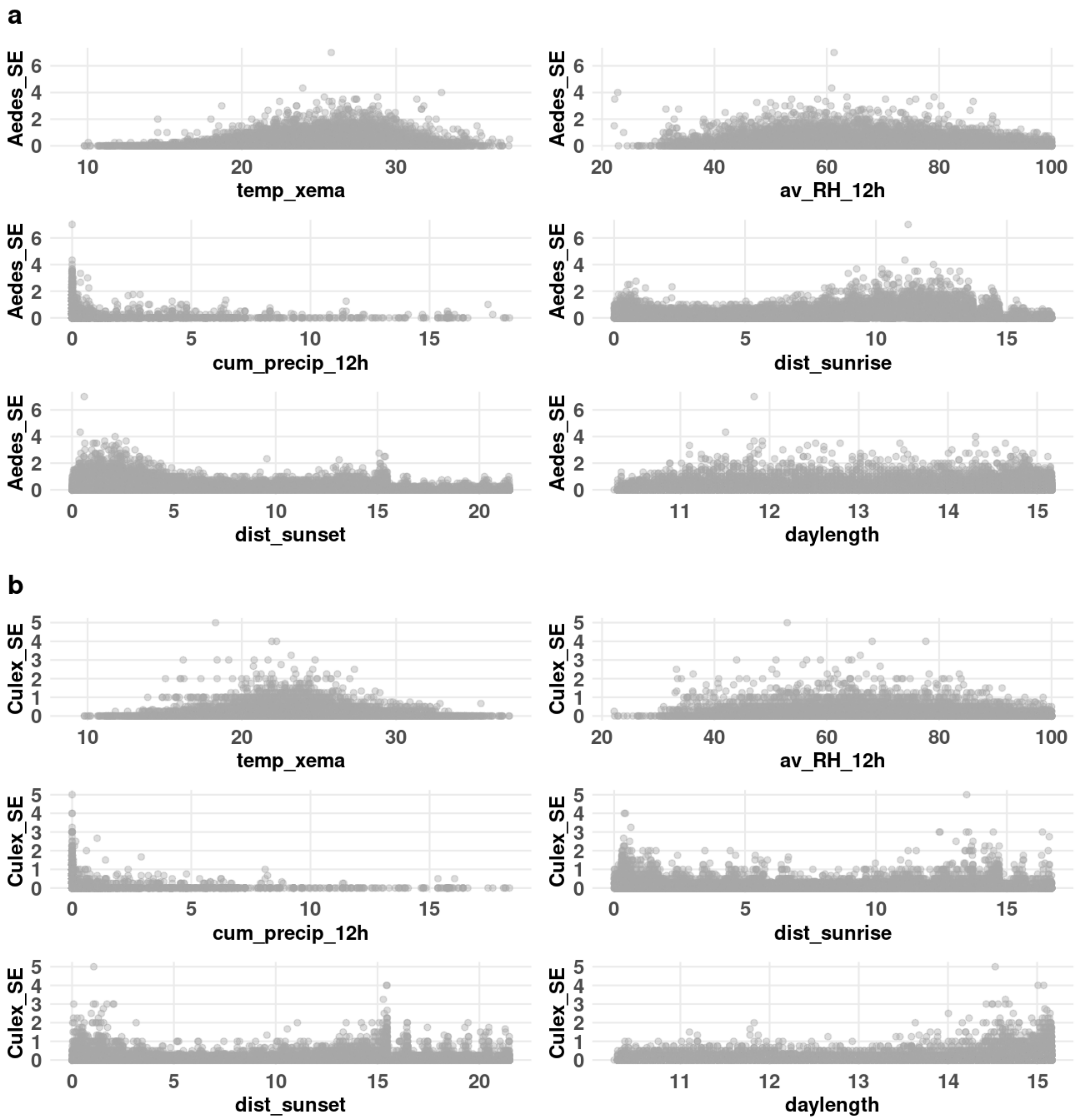

Table S1. List of explanatory variables considered for modeling (name, description and units)

Name	Description	Units
temp_xema	Hourly temperature	ºC
av_temp_24h	Average temperature in the previous 24 hours	ºC
av_temp_12h	Average temperature in the previous 12 hours	ºC
av_temp_6h	Average temperature in the previous 6 hours	ºC
RH_xema	Hourly relative humidity	%
av_RH_24h	Average relative humidity in the previous 24 hours	%
av_RH_12h	Average relative humidity	%
av_RH_6h	Average relative humidity	%
precip_xema	Hourly precipitation	mm
cum_precip_24h	Cumulated precipitation in the previous 24 hours	mm
cum_precip_12h	Cumulated precipitation in the previous 12 hours	mm
cum_precip_6h	Cumulated precipitation in the previous 6 hours	mm
solar_rad_xema	Hourly solar radiation	W/m^2
ADH	Activity Degree Hours	ADH
cum_ADH_24h	Cumulated ADH in the previous 24 hours	ADH
cum_ADH_12h	Cumulated ADH in the previous 12 hours	ADH
cum_ADH_6h	Cumulated ADH in the previous 6 hours	ADH
dist_sunrise	Time distance between the recording event and the sunrise	decimal hours
dist_sunset	Time distance between the recording event and the sunset	decimal hours
daylength	Daylight hours during a day	decimal hours
HOD_utc	Hour of the Day	utc hours
DOY	Day of the Year	days

References

1. Baskerville, G. L. & Emin, P. Rapid estimation of heat accumulation from maximum and minimum temperatures. Ecology **50**, 514–517 (1969).
2. Hachisuca, A. M. M. et al. Growing degree-hours and degree-days in two management zones for each phenological stage of wheat (*Triticum aestivum* L.). Int J Biometeorol **67**, 1169–1183 (2023).
3. Ruybal, J. E., Kramer, L. D. & Kilpatrick, A. M. Geographic variation in the response of *Culex pipiens* life history traits to temperature. Parasites Vectors **9**, 116 (2016).
4. Reinhold, J. M., Lazzari, C. R. & Lahondère, C. Effects of the environmental temperature on *Aedes aegypti* and *Aedes albopictus* mosquitoes: a review. Insects **9**, 158 (2018).
5. González-Pérez, M. I. et al. Field evaluation of an automated mosquito surveillance system which classifies *Aedes* and *Culex* mosquitoes by genus and sex. Parasites Vectors **17**, 97 (2024).
